# Transnasal endoscopic orbital inner wall decompression combined with penetrating keratoplasty for corneal ulcer perforation in Graves’ ophthalmopathy: A case report

**DOI:** 10.1097/MD.0000000000039653

**Published:** 2024-10-04

**Authors:** Pei Wang, Zijian Han, Xiuhong Li, Ru Lin, Hongfeng Yuan

**Affiliations:** a Department of Orbital Surgery, Chongqing Aier Eye Hospital, Chongqing, China; b Aier School Ophthalmology, Central South University, Changsha, Hunan, China.

**Keywords:** corneal ulcer perforation, Graves’ ophthalmopathy, penetrating keratoplasty, transnasal endoscopic orbital inner wall decompression

## Abstract

**Rationale:**

To report a unique case of corneal ulcer perforation associated with a severe manifestation of Graves’ ophthalmopathy that was treated with unilateral transnasal endoscopic orbital inner wall decompression combined with penetrating keratoplasty.

**Patient concerns:**

A 66-year-old male with Graves’ disease experiencing unilateral corneal ulcer perforation, a rarity in medical literature. He presented with a 9-month history of bilateral exophthalmos accompanied by decreased vision, and over the past month, he experienced loss of vision and painful swelling in the right eye (RE).

**Diagnoses:**

After a series of examinations, he was diagnosed with Graves’ ophthalmopathy.

**Interventions:**

Through local anti-infective treatment, the RE corneal ulcer showed no signs of infection. Once the patient’s thyroid function and heart rate were stable and his overall condition was stable, he underwent RE unilateral transnasal endoscopic orbital inner wall decompression combined with penetrating keratoplasty under general anesthesia.

**Outcomes:**

The patient underwent successful unilateral transnasal endoscopic orbital inner wall decompression combined with penetrating keratoplasty, resulting in a significant 4 mm reduction in orbital proptosis and it improved the vision and saved the eyes.

**Lessons:**

Transnasal endoscopic orbital inner wall decompression combined with penetrating keratoplasty is an effective method for treating corneal ulcer perforation caused by Graves’ ophthalmopathy. This method can not only greatly improve the appearance but also save the patient’s eyeball and improve visual function.

## 1. Introduction

Graves’ ophthalmopathy is the most common and quite often severe autoimmune disorder that impairs appearance and visual function, primarily affecting retrobulbar fat, connective tissue, and extraocular muscles.^[1]^ It is the most common orbital disease globally, which has a peak incidence between fourth and sixth decade.^[2,3]^ Its incidence is related to thyroid function, with most patients exhibiting hyperthyroidism, while a minority present with hypothyroidism or normal thyroid function.^[4,5]^ In the course of Graves’ disease, approximately 25% to 50% of patients will develop Graves’ ophthalmopathy, exhibiting symptoms such as lid lag and proptosis, ectropion, exposure keratitis, strabismus, diplopia, compressive optic neuropathy, alterations in visual acuity and potentially blindness.^[6,7]^ Corneal ulcer perforation is one of the most serious complications of Graves’ ophthalmopathy which represents an ophthalmological emergency due to its devastating consequences. They are an important cause of ocular morbidity, leading to a decrease in vision and, in the worst-case scenario, even blindness and loss of the eye. Emergency treatment is mandatory to try to restore the anatomical integrity of the globe, to salvage useful vision as much as possible, and to reduce the possible complications to a minimum.

To our knowledge, we report the first case of severe Graves ophthalmopathy with corneal ulcer perforation and exophthalmos who had successfully undergone transnasal endoscopic orbital inner wall decompression combined with penetrating keratoplasty with subsequent improvement of 4 mm in orbital proptosis, and it improved the vision and saved the eyes. Transnasal endoscopic orbital inner wall decompression combined with penetrating keratoplasty is an effective method to save the visual acuity of patients with corneal ulcer perforation in Graves’ ophthalmopathy.

## 2. Case report

**Figure 1.**
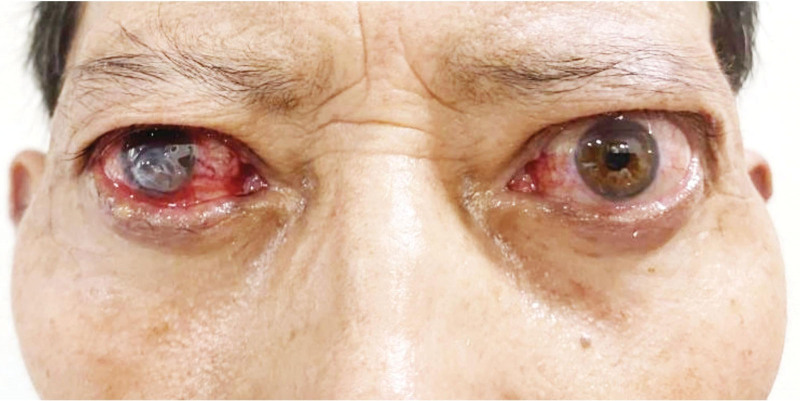
The patient’s appearance on admission: OU (both eyes): exophthalmos, eyelid retraction, incomplete eyelid closure.

**Figure 2.**
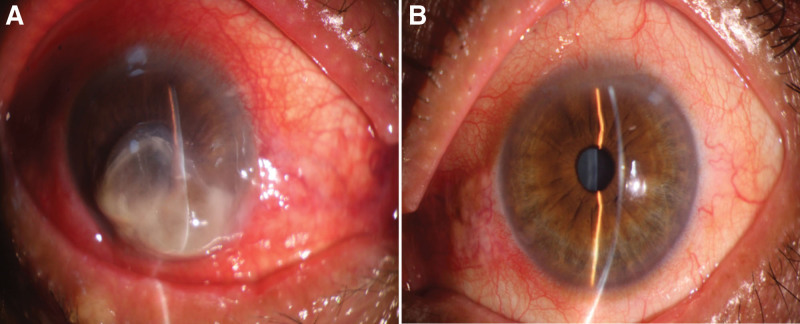
(A) Right eye corneal ulcer with perforation and absence of the anterior chamber at the time of presentation; (B) left eye cornea at the time of presentation.

**Figure 3.**
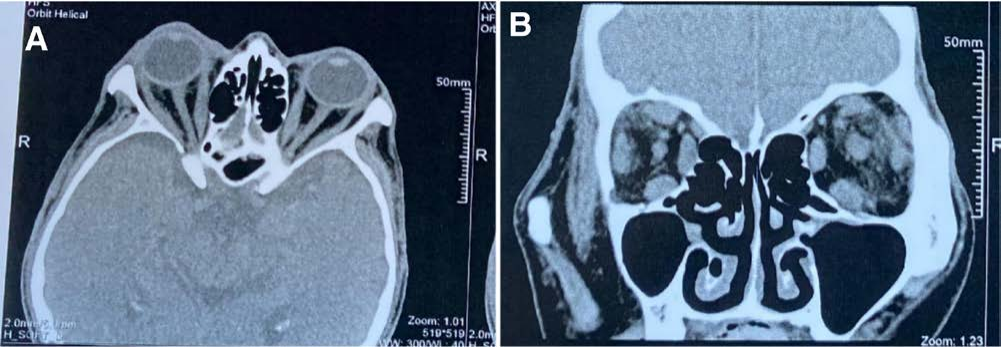
CT scan showing bilateral exophthalmos, bilateral optic nerves, and bilateral thickening of the superior rectus, inferior rectus, medial rectus, and lateral rectus muscles. (A) horizontal position, (B) coronal position.

A 66-year-old male presented with a 9-month history of bilateral exophthalmos accompanied by decreased vision, and over the past month, he experienced loss of vision and painful swelling in the right eye (RE). The patient had a history of hyperthyroidism for 1 year and was irregularly taking methimazole, which he discontinued 1 month before admission. Upon admission, his heart rate was 105 beats per minute. Physical examination showed bilateral marked proptosis (19 mm in RE, 18 mm in left eye [LE]), eyelid retraction, incomplete eyelid closure (Fig. 1). The patient perceived the light perception with the RE and the best corrected visual acuity was 0.5 on LE. The intraocular pressure was normal (14 mm Hg) in RE and (17mm Hg) in the LE. Ophthalmological examination revealed conjunctival redness and chemosis. The upward rotation, downward rotation, and outward rotation of binocular eye movement are limited. Porcelain white opacities and edema can be seen below the cornea of the RE, with a patchy ulcer lesion measuring about 6 × 6 mm in size. Yellow-white exudate can be seen on the ulcer surface, central perforation, disappearance of the anterior chamber, and no obvious pus accumulation in the anterior chamber (Fig. 2). Concurrent cataract was present in the RE. Fundus evaluation of the LE was normal, without suggestive signs of dysthyroid optic neuropathy, whereas the RE could not be evaluated. A computed tomographic scan of the orbits demonstrated bilateral exophthalmos, bilateral optic nerve, bilateral superior rectus, inferior rectus, internal rectus, and external rectus muscle abdominal thickening (Fig. 3). According to the European Group on Graves’ Orbitopathy thyroid eye disease severity scale, the patient was referred with “very serious disease,” due to corneal ulceration perforation. The nervous system was normal.

The patient was admitted to the hospital for further investigations, endocrinologic assessment, and management. Initial blood investigations, including blood count, liver biochemistry, glycemia, serum electrolytes, fasting lipid profile, serum creatinine, blood urea nitrogen, erythrocyte sedimentation rate were normal. The thyroid function and antibody tests showed elevated levels with triiodothyronine: 7.17 nmol/L, free triiodothyronine: 23.81 pmol/L, tetraiodothyronine: 268.59 nmol/L, free tetraiodothyronine: 50.19 pmol/L, thyroid stimulating hormone <0.005 uIU/mL, and thyrotropin receptor antibodies >40 IU/L. The presence of high levels of thyrotropin receptor antibodies autoantibodies suggested an active phase of Graves’ disease.

Post-admission, the patient received high-dose steroid therapy: intravenous sodium succinate methylprednisolone 500 mg for 3 days, then tapered to 250 mg for 3 days, 125 mg for 3 days, and finally 80 mg for 3 days, along with gastroprotective agents, potassium, and calcium supplementation.

**Figure 4.**
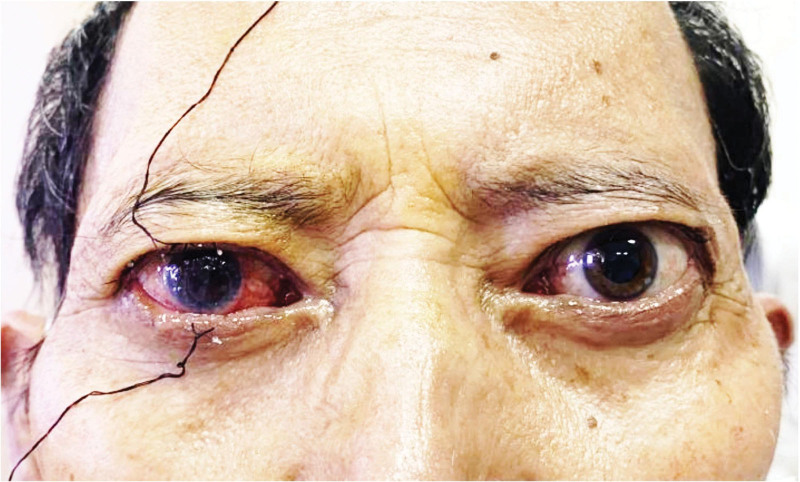
Temporary suturing of upper and lower eyelids of the right eye to reduce exposure during sleep in the early postoperative period.

Additional treatments to protect the cornea included levofloxacin eye drops 4 times a day, tobramycin eye ointment once every night, deproteinized calf blood extract eye gel 3 times a day, sodium hyaluronate eye drops 6 times a day, wearing swimming goggles to reduce exposure and protect the cornea (Fig. 4). Preoperatively, the patient was given oral propylthiouracil and propranolol to control thyroid function, prevent thyroid storm, and reduce heart rate. After 9 days of medication, his thyroid function stabilized, and his heart rate decreased from 105 to 80 beats per minute. The RE corneal ulcer showed no signs of infection. Once the patient’s thyroid function and heart rate were stable and his overall condition was stable, he underwent RE unilateral transnasal endoscopic orbital inner wall decompression combined with penetrating keratoplasty under general anesthesia.

Postoperatively, treatment included anti-infective and anti-corneal rejection measures, and temporary eyelid sutures were used at night to reduce exposure (Fig. 4). Oral hormone therapy included prednisone acetate 40 mg, tapering by 1 tablet per week until discontinuation. Supportive treatments included gastroprotection, potassium, and calcium supplementation. Post-corneal transplantation, anti-rejection therapy included loteprednol etabonate ophthalmic suspension 4 times a day, gatifloxacin eye gel 3 times a day, deproteinized calf blood extract eye gel 3 times a day, cyclosporine eye drops 4 times a day, hyaluronic acid sodium eye drops 6 times a day. Thyroid function was controlled with oral methimazole and levothyroxine sodium tablets.

**Figure 5.**
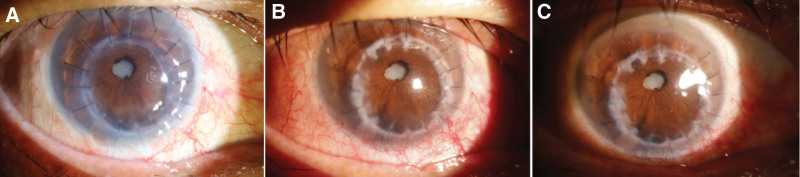
(A) Right eye 1 mo postoperatively, (B) right eye 6 mo postoperatively, (C) right eye 1 y postoperatively.

**Figure 6.**
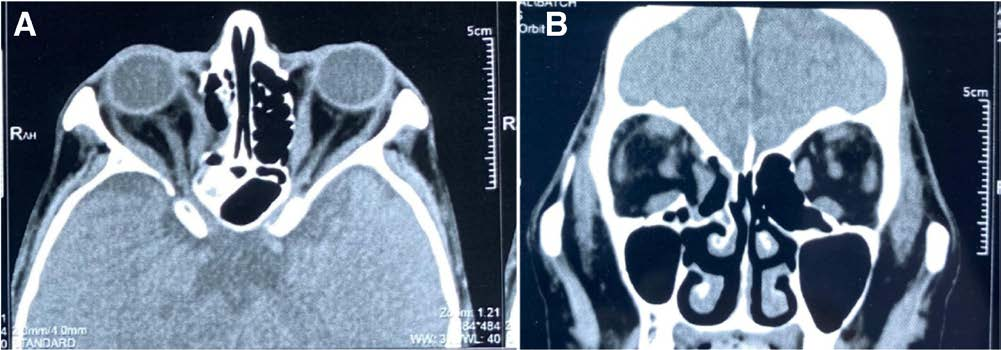
Postoperative orbital CT scan.

At 1-year follow-up (Fig. 5), the transplanted cornea was very stable and visual acuity improved to hand motion/30 cm in the RE and 0.8 in the left eye. The slit lamp shows that the corneal graft is transparent, the corneal graft fits well with the implant bed (Fig. 5). Intraocular pressure was 15 mm Hg in the RE and 16 mm Hg in the LE, with Hertel measurements of 15 mm in the RE and 17 mm in the LE (Fig. 6). The patient delayed the surgical treatment of the cataract in the RE.

## 3. Discussion

Corneal damage is the most dangerous vision-threatening complication of Graves’ ophthalmopathy. In the case of delayed treatment, they can cause permanent visual loss.^[8]^ The severe exposure keratopathy represents an urgent condition, thus decompression surgery could be indicated.^[3,9]^ For patients with Graves’ ophthalmopathy demonstrating no response to immunosuppressive therapy, orbital decompression is a key element of treatment and subsequent rehabilitation.^[10,11]^ The primary goal of orbital decompression is to increase the orbit space to tolerate the increased volume of orbital tissue generated by the autoimmune activation of orbital fibroblast.

Endoscopic orbital decompression was first reported in 1990 with axial proptosis being reduced by a mean of 5.7 mm when combined with lateral orbitotomy and 4.7 mm when decompressed by an endoscopic approach alone.^[12,13]^ Wee et al^[14]^ reported endoscopic orbital decompressions with an average improvement of 4.4 mm in orbital proptosis. Neugebauer et al^[15]^ reported a mean recession of 3.0 mm of 42 orbits. She et al^[16]^ found that transnasal endoscopic orbital decompression surgery is obviously beneficial in relieving exposure keratitis and can significantly improve proptosis, intraocular pressure, and visual acuity with favorable cosmetic results and rare complications.^[16]^ However, transnasal orbital decompression surgery often more needed an experienced endoscopist to improve surgical safety.

Comparing the endoscopic transnasal orbital decompression with other traditional methods, the intranasal endoscope has significantly improved anatomic visualization of the nose and paranasal sinuses.^[16]^ Thus, it permits a more complete medial orbital decompression. This has allowed for a transnasal decompression of the medial and inferior orbital walls that compared well with traditional methods. If in the hands of an experienced sinus endoscopist, transnasal orbital decompression appears to be a safe procedure that can be performed with a minimum of morbidity.^[13]^ Lots of research have indicated the efficacy and relative safety of orbital decompression.^[17]^ Endoscopes also permit a maximal posterior orbital decompression at the orbital apex, an area often not fully accessible via transantral routes. This provides optimal decompression of the optic nerve in cases of optic neuropathy.^[16,17]^

This clinical case is of interest because of a severe course of refractory Graves’ ophthalmopathy, which resulted in both corneal ulcer perforation and exophthalmos. This patient is due to exophthalmos caused by Graves’ ophthalmopathy, resulting in inability to close the eyelids, leading to exposure keratitis and perforation after corneal ulcer. Corneal perforation is an emergency in ophthalmology, which needs emergency surgical repair to prevent endophthalmitis caused by bacterial infection. Once endophthalmitis occurs, most of them will lead to enucleation. Therefore, the urgent treatment for patients is to repair corneal perforation. The best and most likely way to restore vision is corneal transplantation.

However, the patient is the eyelid insufficiency caused by high eyeball protrusion and the perforation caused by exposed corneal ulcer. If corneal transplantation alone cannot solve the eyelid insufficiency caused by eyeball protrusion, the exposed corneal ulcer will appear in the corneal graft and the perforation will occur again. Therefore, it is necessary to do orbital decompression before corneal transplantation. Moreover, orbital decompression surgery can reduce intraorbital pressure and reduce the possibility of atrophy caused by optic nerve compression.

However, if orbital decompression is performed first and corneal transplantation is performed after the eyelids can be closed, 2 operations are required, which increases the risk of anesthesia, the probability of intraocular infection and hyperthyroidism crisis, and the economic burden. Combined corneal transplantation to reduce the above risks.

The main difficulties and risks of this combined operation are: how to control thyroid function in the shortest time and reduce the risk of hyperthyroidism crisis during operation. The area of corneal perforation and ulcer is large, the lower part is close to the corneal limbus, and the eye is in the active stage of inflammation. How to reduce the probability of postoperative rejection, whether concurrent cataract needs to be treated at the same time, and how to avoid secondary glaucoma after long-term disappearance of anterior chamber. In theory, the balanced decompression of the inner and outer walls of the orbit is required, but the conventional decompression methods will compress the eyeball during the operation, which will have an impact on the corneal transplantation. Whether the simple nasal endoscopic orbital wall decompression can achieve sufficient decompression effect and whether the eyelids can be completely closed after operation. Through multi-disciplinary detailed discussion, we finally adopted the way of corneal transplantation combined with nasal endoscopic orbital wall decompression to successfully complete the operation.

At present, the effect is good. The exophthalmos of the patient’s RE has improved significantly. It has retreated from 19 mm before operation to 14 mm now, and the closed eye has returned to normal. However, for patients, it is still necessary to reasonably control thyroid function for a long time, inhibit rejection, and promote the transparency of corneal grafts. Cataract surgery is also needed to restore vision in the later stage.

Although similar combined surgery has not been reported yet, we have also achieved breakthroughs and innovations, making the surgery very successful. Thyroid-associated ophthalmopathy can be treated and early detection and treatment will not lead to blindness.

## Author contributions

**Conceptualization:** Pei Wang, Hongfeng Yuan.

**Data curation:** Pei Wang, Zijian Han, Xiuhong Li, Ru Lin, Hongfeng Yuan.

**Methodology:** Pei Wang, Xiuhong Li, Ru Lin.

**Writing—original draft:** Pei Wang.

**Writing—review & editing:** Pei Wang, Hongfeng Yuan.

**Project administration:** Zijian Han.
